# Anti-CASPR2 Antibody-Associated Autoimmune Encephalitis Presenting as Refractory Seizures

**DOI:** 10.7759/cureus.64317

**Published:** 2024-07-11

**Authors:** Nivedha Balaji, Aleksandra Ignatowicz, Aarushi Kalra, Rami Mansour, Vaishali Jadhav

**Affiliations:** 1 Internal Medicine, Northeast Georgia Medical Center Gainsville, Gainesville, USA; 2 Internal Medicine, Philadelphia College of Osteopathic Medicine, Suwanee, USA

**Keywords:** paraneoplastic syndrome, encephalitides, recurrent seizures, encephalitis, autoimmune encephalitis

## Abstract

Autoimmune encephalitis (AE) is a rare immune-mediated disorder comprised of non-infectious neuroinflammatory disease processes. Clinical presentation overlaps with a broad range of neurodegenerative disorders and infectious encephalitis; therefore, AE remains a diagnosis of exclusion. Patients may present with nonspecific symptoms such as psychiatric disturbances, cognitive deficits, seizures, movement disorders, and confusion. Prompt diagnosis and management are necessary for patients with AE to decrease mortality and improve quality of life. First-line therapy includes immunosuppression with corticosteroids, intravenous immunoglobulin, and plasmapheresis. We report the case of an 86-year-old female with a medical history of Parkinson’s disease who presented with nonspecific seizure-like activity and was diagnosed with AE.

## Introduction

Autoimmune encephalitis (AE) is a complex condition where the immune system mistakenly attacks the brain, leading to inflammation. This can result in a range of neuropsychiatric symptoms due to specific autoantibodies targeting neuronal cell surface antigens [1]. Estimating the prevalence of AE is difficult because of its diverse clinical presentations and relatively recent recognition in the 20th century. It is believed to affect around one in 100,000 people annually [2]. Greater awareness and the identification of new autoantibodies have led to more frequent diagnoses [3]. The symptoms of AE vary widely, complicating diagnosis and rapid development over a few days to weeks. Common signs include psychiatric disturbances (such as anxiety, psychosis, and behavioral changes), cognitive deficits, seizures, movement disorders, autonomic instability, and altered consciousness [1]. The causes of AE are varied. It can occur as a paraneoplastic syndrome, where an immune response is triggered by a tumor expressing neuronal antigens [4]. Non-paraneoplastic cases are often associated with viral infections, genetic factors, or other autoimmune diseases [5]. Several key autoantibodies involved in AE include those against NMDA receptors, LGI1, CASPR2, and AMPA receptors [6]. 

## Case presentation

The patient, an 86-year-old female with a medical history of Parkinson's disease, recurrent urinary tract infections, and seizure-like episodes, presented to the emergency department (ED) after experiencing recurrent seizure-like activity. The patient was found to be minimally responsive, with twitching of her jaw and upper extremities, rigidity of her neck and jaw pulling her head to the left side, loss of bladder function, and tongue biting, followed by a post-ictal state. 

Of note, the patient had two prior admissions with similar complaints of seizure-like activity. During these hospitalizations, she had a diagnostic workup that included computed tomography (CT) brain, magnetic resonance imaging (MRI) brain, transthoracic echocardiogram (TTE), and electroencephalography (EEG), which were all unremarkable. She was diagnosed with non-epileptic spells and discharged without anti-epileptic medications. 

In the ED, her vital signs were stable. A physical examination was significant for crackles in the right lower lung field. Significant laboratory findings on presentation are noted in Table 1. 

**Table 1:** Patient’s significant laboratory values upon presentation

Component	Patient’s values	Normal range
White blood cell	12.6 x 10^9^/L	4.0 – 10.0 x 10^9/^L
Hemoglobin	10.7 g/dL	12 – 15 g/dL
Sodium	134 mmol/L	135 – 145 mmol/L

**Figure 1:**
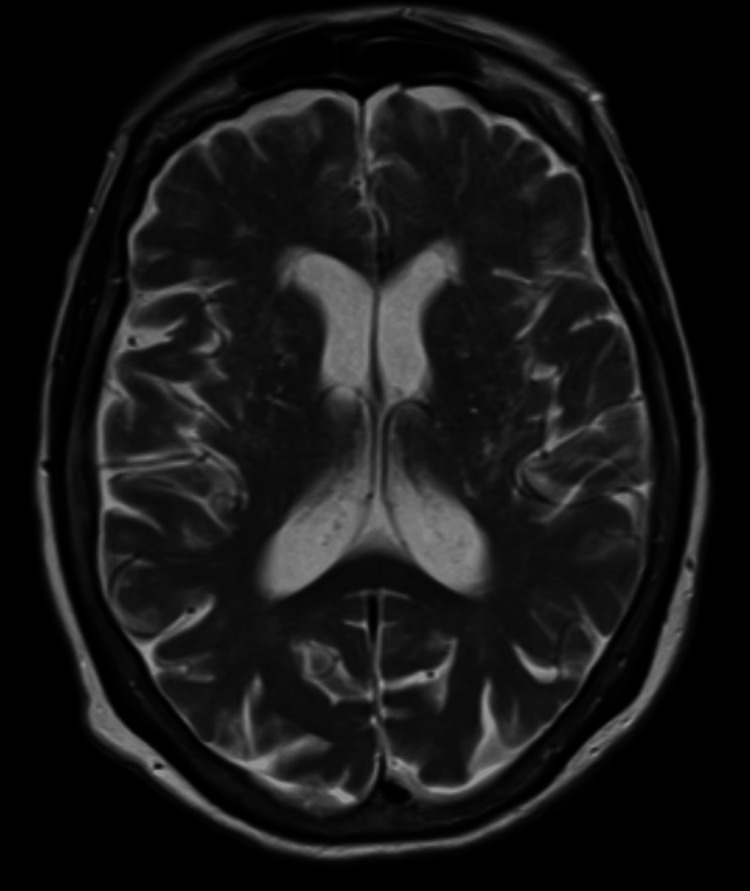
An MRI of the brain shows no acute abnormality

A chest radiograph showed right lung base densities, which were concerning for pneumonia. She was admitted to the inpatient medicine service for treatment of community-acquired pneumonia and an epilepsy workup. She was started on levetiracetam for seizure-like activity and ceftriaxone with doxycycline for community-acquired pneumonia. Neurology and psychiatry were consulted. The CT scan of the brain and computed tomography angiography (CTA) of the head and neck were unremarkable. The initial EEG shown in Figure 1 revealed seizure-like activity captured as non-epileptic spells. On follow-up video monitoring, she was noted to have three tonic-clonic seizures. However, on the continuous EEG that accompanied the video monitor, there was an excessively low-voltage beta background without epileptiform activity. Such findings were suggestive of atypical encephalopathy. Due to the use of a secondary service for EEG interpretation, only EEG reports were available for review; the EEG images were not available. Psychiatry started the patient on quetiapine and sertraline for concerns about anxiety being a trigger for the seizure-like activity. She was subsequently switched from levetiracetam to valproic acid due to persistent seizure-like spells on levetiracetam. Valproic acid levels were noted to be therapeutic. 

Due to ongoing seizures not managed with anti-epileptic drugs, a lumbar puncture was performed to pursue an infectious workup. The cerebrospinal fluid (CSF) fluid analysis revealed glucose levels of 42 mg/dL (normal range: 40-80 mg/dL), elevated protein of 53.9 mg/dL (normal range: 15.0-45.0 mg/dL), white blood cell (WBC) count 6 per mm^3 (normal range: 0-5 per mm^3), and lymphocytes of 12%. The CSF was negative for growth on cultures and negative for cryptococcus and meningitis etiologies. However, CSF revealed positive levels for CASPR2 antibodies, which was consistent with AE. Subsequently, an MRI of the brain (Figure 1) was performed and it was unremarkable.

Due to the association of CASPR2 with malignancies, she underwent a CT of the chest, abdomen, and pelvis to rule out any masses or adenopathy; however, the results were negative for neoplastic etiologies. The patient underwent five sessions of plasmapheresis during her hospitalization. The patient was later discharged to a skilled nursing facility without further seizure-like episodes. 

Unfortunately, the patient returned approximately 13 months later with status epilepticus and respiratory failure secondary to aspiration pneumonia requiring intubation. No acute intracranial etiologies were noted on brain imaging at that time; however, it is unclear whether the hospitalization was a relapse of previous autoimmune encephalitis or an unrelated event. The patient was transitioned to hospice and passed away during that hospitalization. 

## Discussion

Acute encephalitis was previously seen as a result of infectious etiologies; however, there is a rise in autoimmune cases with the formation of antibodies against neuronal intracellular antigens, ion channels, cell surface proteins, and synaptic receptors [7]. Several of the tested antibodies include Hu, Ma1, GAD antigens, NMDA, AMPA, mGluR5, dopamine 2 or GABA A or B receptors, LGI1, CASPR2, DPPX, MOG, aquaporin 4, and GQ1b, to name a few. Depending on the specific antibody, encephalitis or encephalomyelitis can occur in a particular region, such as the limbic structures with hypothalamic and mesencephalic involvement, the basal ganglia, the cerebellum with concerns for degeneration, and the brainstem. It can also be associated with certain malignancies or disease processes, such as ovarian teratomas [8]. 

According to Graus et al., the approach to diagnosing AE has changed to account for other factors such as autoantibody screening, clinical presentation, findings of normal imaging, and association with other diseases or autoimmune processes. One of the limitations of using antibody testing as a primary factor in the diagnostic approach was the lack of accessibility for antibody screening in specific hospital systems and the prolonged time to obtain results. There were also conflicting data suggesting that a positive or negative screen might not indicate an immune-mediated process. Subsequently, the proposed diagnostic criteria now consider the presence of the following: subacute onset within three months with concerns for memory deficits, psychiatric symptoms, altered mental status, exclusion of other causes, new focal CNS findings, unexplained seizures, CSF pleocytosis, or positive MRI findings. Unfortunately, MRI results can be normal or nonspecific in the early stages. An EEG can be inconclusive at times as well; however, it can show subclinical seizures or non-convulsive status epilepticus [1, 9]. Patients may also experience myoclonic activity without correlating EEG findings, which is suggestive of atypical encephalopathy [9]. Finally, alternative causes must be excluded, including vascular disorders, neurodegenerative diseases, rheumatologic disorders, toxic-metabolic disturbances, and infectious causes with viruses, bacteria, or fungi [1, 9]. 

There is a spectrum of AE classified according to the specific autoantibodies, lack of antibodies, onset of presentation, and affected area of the CNS. In autoimmune limbic encephalitis, the diagnostic approach was modified to account for bilateral involvement of the medial temporal lobes on imaging with positive autoantibodies and either CSF pleocytosis or abnormal EEG in the temporal lobes [1]. An MRI of the brain can reveal T2-weighted fluid-attenuated inversion recovery (FLAIR) studies in the previously mentioned temporal lobes and limbic systems [10]. Of the antibodies, the neuronal cell surface antibodies, LGI1, GABA, AMPA, and CASPR2, and the onconeural antibodies, Hu and Ma2, are more frequently associated with limbic encephalitis [9, 10]. Anti-Hu or ANNA-1 and Ma2 are intracellular autoantibodies with strong associations with oncologic etiologies, commonly testicular germ cell tumors, both non-small cell and small cell lung carcinoma, or neuroblastoma [11]. This patient was diagnosed with CASPR2 autoimmune encephalopathy. Autoantibodies are produced against CASPR2, a neuronal cell surface antigen, later manifesting as limbic encephalopathy, autoimmune epilepsy, cerebellar dysfunction, or peripheral nerve excitability-causing neuropathy. There is an oncologic association with most common thymomas and several reported melanomas, although it’s a rare finding [11,12]. CASPR2-mediated encephalitis is treated with immunotherapies and anti-epileptic medications. In a study by Lancaster et al., a patient with CASPR2 encephalitis completed a course of high-dose steroids with methylprednisolone, followed by a slow prednisone taper and a four-week cycle of rituximab [5]. Studies recommend a prolonged corticosteroid taper over 18 months to decrease the risk of relapses [12]. According to Binks et al., the rate of relapse is variable; however, the clinical course is usually monophasic in non-paraneoplastic cases [12]. 

Acute disseminated encephalomyelitis does not have specific biomarkers; however, imaging can reveal multiple abnormalities in the supratentorial white matter, basal ganglia, brainstem, cerebellum, and spinal cord on MRI within three months of symptom onset. Bickerstaff’s brainstem encephalitis has a subacute onset within less than four weeks with impaired consciousness, bilateral external ophthalmoplegia, and ataxia. This is often preceded by an infectious etiology and positive anti-GQ1b antibodies. Magnetic resonance imaging can be normal or show a brainstem abnormality [1, 13]. In Hashimoto’s encephalopathy, patients can present with seizures, hallucinations, or stroke-like symptoms and may have subclinical or mild thyroid disease with positive thyroid antibodies. Imaging is often normal or nonspecific, with findings consistent with leukoencephalopathy, and neuronal antibodies are expected to be absent in both the serum and CSF [2, 10]. In some instances, there is an antibody-negative autoimmune encephalitis. These patients will show rapid progression of neurologic symptoms and an absence of autoantibodies in both the serum and CSF. Diagnosis will depend on the exclusion of other autoimmune encephalitis syndromes, CSF pleocytosis, or a negative brain biopsy of other inflammatory causes of disorders. MRI findings can suggest AE or show signs of multifocal white matter demyelination [1, 14]. 

The antibody prevalence in epilepsy and encephalopathy (APE2) criteria is a scoring system that can be used to predict neural-specific antibody seropositivity. The APE2 criteria assign one point for new-onset seizure activity or mental status change, one point for neuropsychiatric changes, one point for autonomic dysfunction, two points for viral prodromal symptoms, three points for faciobrachial dystonic seizures, two points for facial dyskinesia, two points for seizures refractory to at least two antiepileptic medications, two points for CSF findings consistent with inflammation, two points for an MRI of the brain suggesting encephalitis, and two points for systemic malignancy. Patients with epilepsy of unknown etiology and an APE2 score of at least four have a sensitivity of 98% and a specificity of 85% for autoimmune etiology. An APE2 score greater than six has a specificity of 100% for autoimmune etiology-induced epilepsy [15]. 

Prompt treatment of AE is recommended to decrease mortality, decrease long-term complications, and improve quality of life with improved cognitive impairment and seizure control. Depending on the etiology, immunosuppressive therapies are mainly used with IV corticosteroids, pulsed methylprednisolone for five days, intravenous immunoglobulin (IVIG), and plasmapheresis as the initial therapies. Second- and third-line therapy is recommended if there is an inefficient response with the first-line therapies or relapse of symptoms with rituximab and cyclophosphamide and tocilizumab and intrathecal methotrexate, respectively. Maintenance therapy can be continued for an additional six months with IVIG, methylprednisolone, rituximab, oral prednisone, azathioprine, and mycophenolate [2, 9]. However, responsiveness to immunosuppression must be determined, as autoantibodies to intracellular antigens compared to neuronal surface antigens respond poorly to immunotherapy due to immunopathology [8]. 

According to several studies, recovery may take up to two years with full resolution or near resolution of symptoms. In certain cases, maintenance therapy needs to be continued for one to two years to prevent relapse [9, 16]. 

There is concern about whether the patient was readmitted for relapse of her AE despite the completion of primary treatment with plasmapheresis. She was discharged on antiepileptic drugs without a prolonged course of steroids or follow-up for the need for secondary treatment or maintenance therapy. The risks and benefits of using maintenance therapies for prolonged periods of time would need to be another consideration and discussion with the patients accordingly. With her history of refractory seizures despite antiepileptic drugs, there is concern about whether an additional prolonged, steroid course would have been recommended for this patient. She did not undergo further AE workup after the last admission for status epilepticus, unfortunately, so the authors are unable to ascertain whether the seizures were a relapse of her condition within a two-year period. This concern brings up the question of an appropriate post-treatment protocol in patients with AE to ensure complete resolution of the condition and prevent relapse, although some studies noted that CASPR2 AE was often a monophasic course. 

## Conclusions

In conclusion, this case report highlights the importance of maintaining clinical vigilance when encountering patients with nonspecific symptoms. It reiterates the need to maintain a broad differential, consideration for a prompt diagnostic workup, and initiation of management for patients with AE. Patients with AE present with several non-specific symptoms; hence, a detailed history, physical examination, and diagnostic testing are needed to appropriately identify the disease and exclude relevant pathogens. Diagnostic workup, including ancillary tests such as MRI, EEG, and definitive testing with a lumbar puncture showing antibodies, can diagnose AE. In patients with AE, due to the risk of malignancy, workup should also check for specific tumors. Prompt diagnosis can help deliver immunosuppressive therapy in a timely manner. Management with corticosteroids and/or plasmapheresis remains the first-line of treatment. 
